# Detection of the *EGFR* G719S Mutation in Non-small Cell Lung Cancer Using Droplet Digital PCR

**DOI:** 10.3389/fmed.2020.594900

**Published:** 2020-11-13

**Authors:** Margalida Esteva-Socias, Mónica Enver-Sumaya, Cristina Gómez-Bellvert, Mónica Guillot, Aitor Azkárate, Raquel Marsé, Úrsula Sastre, Ana Blasco, Silvia Calabuig-Fariñas, Víctor José Asensio, Josefa Terrasa, Antònia Obrador-Hevia

**Affiliations:** ^1^Centro de Investigación Biomédica en Red in Respiratory Diseases (CIBERES), Plataforma Biobanco Pulmonar CIBERES, Hospital Universitari Son Espases, Palma, Spain; ^2^Grupo de Inflamación, reparación y cáncer en enfermedades respiratorias, Institut d'Investigació Sanitària de les Illes Balears (IdISBa), Hospital Universitari Son Espases, Palma, Spain; ^3^Group of Advanced Therapies and Biomarkers in Clinical Oncology, Institut d'Investigació Sanitària de les Illes Balears (IdISBa), Hospital Universitari Son Espases, Palma, Spain; ^4^Department of Pathology, Hospital Universitari Son Espases, Palma, Spain; ^5^Department of Oncology, Hospital Universitari Son Espases, Palma, Spain; ^6^Grupo de Enfermedad Oncológica Peritoneal, Institut d'Investigació Sanitária de les Illes Balears (IdISBa), Hospital Universitari Son Espases, Palma, Spain; ^7^Centro de Investigación Biomédica en Red Cáncer (CIBERONC), Madrid, Spain; ^8^Department of Medical Oncology, Hospital General Universitario de Valencia, Valencia, Spain; ^9^Molecular Oncology Laboratory, General University Hospital Research Foundation, Valencia, Spain; ^10^Mixed Unit TRIAL CIPF-FIHGUV, Valencia, Spain; ^11^Department of Pathology, Universitat de València, Valencia, Spain; ^12^Molecular Diagnosis and Clinical Genetics Unit, Hospital Universitari Son Espases, Palma, Spain; ^13^Grupo Genòmica de la Salut, Institut d'Investigació Sanitària de les Illes Balears (IdISBa), Hospital Universitari Son Espases, Palma, Spain

**Keywords:** EGFR, liquid biopsy, G719S, lung cancer, ddPCR = droplet digital PCR, non-small cell lung cancer

## Abstract

**Objectives:** The main objectives of the study were (1) to set-up a droplet digital PCR (ddPCR) assay for the non-invasive detection of G719S EGFR mutation in NSCLC patients; (2) to determine the limits of detection of the ddPCR assay for G719S mutation and (3) to compare COBAS® and ddPCR System for G719S quantification in plasma.

**Materials and Methods:** Blood samples were collected from 22 patients diagnosed with advanced NSCLC. Then, plasma ctDNA was extracted with the Qiagen Circulating Nucleic Acids kit and quantified by QuantiFluor® dsDNA System. The mutational study of EGFR was carried out by digital droplet PCR (ddPCR) with the QX200 Droplet Digital PCR System with specific probes and primers.

**Results:** We observed the lowest percentage of G719S mutant allele could be detected in a wildtype background was 0.058%. In the specificity analysis, low levels of G719S mutation were detected in healthy volunteers with a peak of 21.65 mutant copies per milliliter of plasma and 6.35 MAFs. In those patients whose tissue biopsy was positive for G719S mutation, mutant alleles could also be detected in plasma using both ddPCR and COBAS® System. Finally, when mutational status was studied using both genotyping techniques, higher mutant copies/ml and higher mutant allele fraction (MAF) correlated with higher Semiquantitative Index obtained by COBAS®.

**Conclusions:** Although tissue biopsies cannot be replaced due to the large amount of information they provide regarding tumor type and structure, liquid biopsy and ddPCR represents a new promising strategy for genetic analysis of tumors from plasma samples. In the present study, G719S mutation was detected in a highly sensitive manner, allowing its monitorization with a non-invasive technique.

## Introduction

Cancer is the second leading cause of death in developed countries (1) and lung cancer is the leading cause of cancer death in Europe. Metastatic lung cancer patients surviving for 5 years are <15% (2). Eighty-seven percentage of all cases of lung cancer are non-small cell lung cancers (NSCLC). In order to improve survival of patients, research has focused on understanding the biology of tumors to develop targeted therapies and personalized medicine.

In NSCLC several recurrent mutations in genes involved in proliferation, apoptosis, cell survival and angiogenesis have been reported. One of the most important deregulated genes in NSCLC is *EGFR* (Epidermal Growth Factor Receptor). Genetic analysis of NSCLC tumors, especially adenocarcinomas, revealed that around 17% of them harbored *EGFR* mutations (3). About 90% of these mutations are small deletions in 5 amino acids from codon 746 to 750 of exon 19 or missense mutations at codon 858 of exon 21 (4, 5). Moreover, less frequent mutations have been found like the mutation within the phosphate-binding loop (P-loop) that comprises part of the ATP-binding pocket which replaces Gly719 with Ser (G719S) (6, 7). All of these mutations produce a gain of function. NSCLC cells become dependent on this aberrant signaling and inhibition with tyrosine kinase inhibitors (TKIs) specific for *EGFR* like erlotinib and gefitinib, among others, drive to cell death through intrinsic apoptosis (8, 9) achieving long survival rates over 2 years in some cases. Unfortunately, TKIs effects are limited because of resistance occurrence due to several mechanisms, one of which being secondary resistance mutations in *EGFR* (normally T790M mutation) (10, 11).

Screening for mutations in *EGFR* follows two objectives: selection of patients for treatment with TKIs and detection of resistance mechanisms. Tumor biopsies are the gold standard method for detecting these mutations. However, they are spatially and temporary limited due to: biopsies are invasive, often difficult to perform, do not reflect the entire tumor or different metastasis (12, 13). Liquid biopsy for the study of circulating tumor DNA (ctDNA) is being developed to overcome some of these limitations (14, 15). In this study, we have developed a method for detecting G719S mutation in liquid biopsy by means of digital droplet PCR technology.

## Methods

### Patients

Twenty-two patients diagnosed with advanced or metastatic non-small cell lung cancer were recruited to the study from Hospital Universitari Son Espases (HUSE) and Hospital General Universitari de València (HGUVA) from October 2015 to September 2016. The study was approved by the Clinical Research Ethics Committee of the Balearic Islands and Valencia and a written informed consent was acquired from all patients for specimen collection, clinical information collection and biomarker analysis in tissue and plasma samples. Clinical and pathological features of patients enrolled are provided in Table 1. Patients were eligible for the study according to the following selection criteria: histological confirmation of advanced NSCLC, functional state 0–2 according to Performance Status (PS) and patients of both sexes, aged over 18 and belonging to any ethnic group. Pregnant or breastfeeding women and patients with other antecedent of solid or hematological tumors in the previous 5 years, except for basal cell carcinoma, were excluded. Six healthy volunteers with no known significant health problems were also included in the study.

**Table 1:** Outline of clinical and pathological features of patients.

**Variable**	**Total (±SD)**	**Percentage (%)**
Age (years)	67 ± 14	**–**
**Gender**		
Male	9	41
Female	13	59
Smoking habit	12	55
**Pack-year**		
<20	4	33
>20	8	77
**Stage**		
IVA	9	41
IVB	13	59
**NSCLC**		
Primary	20	91
Secondary	2	9
**Treatment**		
None	5	23
First-line chemotherapy	7	32
Second-line chemotherapy	3	14
Third-line chemotherapy	2	9
TKIs^a^	9	40

a *All patients treated with TKIs had at some point received conventional chemotherapy (before or after) based on cisplatin or carboplatin cycles combined with pemetrexed, paclitaxel, or docetaxel*.

*Cumulative smoking exposure was determined in terms of pack-years by multiplying the number of years smoked by the average number of packs per day*.

Tumor genotyping of *EGFR* mutation was carried out in the HUSE Pathology Department using DNA extracted from formalin-fixed paraffin-embedded (FFPE) tissue and COBAS® 4800 system (Roche).

### Plasma Collection and DNA Extraction

Blood samples were collected in Vacutainer EDTA tubes and immediately separated into plasma by centrifugation at 3,000 rpm for 10 min at room temperature. Plasma samples were stored in 2 mL aliquots at −80°C until ctDNA extraction. We analyzed the samples corresponding to the dates of baseline, first month and third month after treatment and progressive disease.

ctDNA extraction was performed using 2 mL of plasma from each patient using the Qiagen Circulating Nucleic Acids kit (Qiagen, Hilden, Germany) following the manufacturer's recommendations. Extracted ctDNA from each plasma sample was twice eluted in 100 and 50 μL of AVE elution buffer and stored at −20°C until mutation profiling. Quantification was performed by QuantiFluor® dsDNA System (Promega Corporation, Alcobendas, Madrid) using 4.8 μL of sample diluted 1/50 with TE 1x buffer (included in kit) following the manufacturer's instructions. Fluorescence measurement was carried out by multiple well spectrophotometer (BioTek, Winooski, VT, USA) and DNA concentrations were obtained in ng/μL.

### G719S Mutation Detection in ctDNA

Mutation analysis was carried out with droplet digital PCR (ddPCR) System (Bio-Rad). The reaction mix was prepared using 10 μL from SuperMix for Probes without dUTP (Bio-Rad), 1 μL from each probe at 5 μM (HEX for the WT and FAM for the mutant), 1 μL from each primer at 9 μM (Supplementary Table 1), and 6 μL from DNA extraction (concentration varies according to the sample used). A total of 20 μL were charged in the QX200 droplet generator (Bio-Rad) and immediately transferred to a 96-well plate through and amplified in a conventional thermal cycler. After PCR reaction, plate was placed in the QX200 reader (Bio-Rad) and data analysis was carried out with Quantasoft™ Analysis Pro Software 1.0.596 (Bio-Rad). For each sample, detected droplets from triplicates were merged into 1 metawell. Wild-type and mutant allele concentrations present in the original blood samples were calculated using the following algorithm:

$$\begin{array}{l} C_{ORIG}=\frac{20\;x\;C_{I}\;x\;V_{E}}{V_{P}\;x\;V_{O}} \end{array}$$

where C_ORIG_ represents mutant allele concentration in the original plasma sample in copies/mL, V_E_ is the elution volume of ctDNA generated by the DNA extraction (100 mL); V_P_ is the volume of elution of DNA used in the PCR reaction (μL); V_O_ is the volume of plasma used to extract ctDNA (2 mL). The value of 20 located in the numerator of the equation corresponds to the final volume PCR mix, which was 20 μL.

Mutant-allele fraction (MAF) data was also calculated as (16):

$$\begin{array}{l} MAF=\frac{mutant\;reads}{mutant\;reads+wild-type\;reads} \end{array}$$

### Limit of Detection Calculation

To determine the limit of detection (LOD), DNA template extracted from FFPE G719S mutant were serially diluted with wild-type DNA at levels of 0.003, 0.03, 0.3, 3, and 30% using a total of 25 ng per well. The LOD was defined as the MAF or the lowest % of mutant allele that can be reliably detected (17).

### Statistical Analysis

Data analysis was carried out with the IBM SPSS Statistics 22 software and the graphical representation was performed with GraphPad Prism 5. For the comparative analysis of COBAS and ddPCR results, we applied the Kappa statistic to determine the measure of agreement between variables.

## Results

### Validation and Sensitivity of G719S Testing With ddPCR

**Figure 1:**
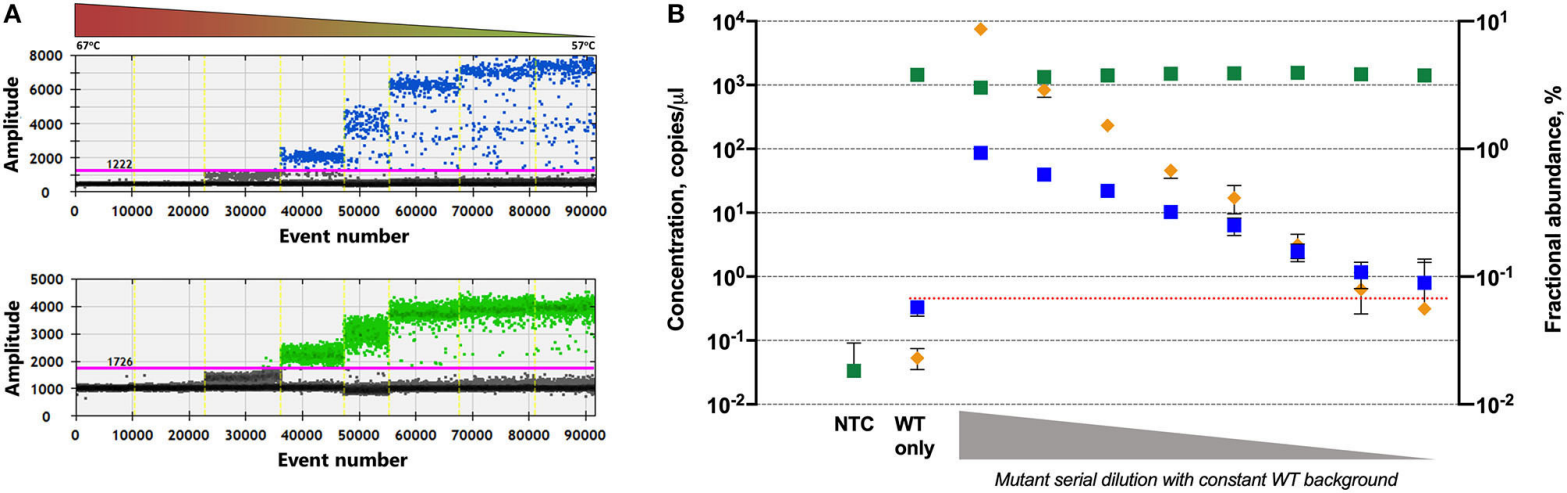
Validation assays for G719S mutation detection in plasma samples. **(A)** Temperature gradient to determine the optimum annealing temperature; mutant positive events (top) and wild-type positive events (bottom) across the thermal gradient (57–67°C). **(B)** Two-fold dilution series of mutant DNA into wild-type DNA: concentration is shown in copies per microliter for mutant (blue) and wild-type (green) events and fractional abundance in percentage (orange). Error bars show 95% CI.

G719S ddPCR assay was tested across an annealing temperature gradient to optimize thermocycling conditions. In order to perform it, we used DNA from positive tumor biopsies, confirmed by COBAS®4800 System, of patients diagnosed with advanced stage of NSCLC. The temperature range studied came from 57 to 67°C and the experiment was repeated twice. Decreasing annealing temperature increased FAM amplitude of the mutant probe and showed a good separation between the four droplet groups to plateau at 57.8°C, allowing clear identification and quantification of both mutant and wild-type droplet groups (Figure 1A).

To calculate empirically the limit of detection we serially diluted positive mutant control DNA (from FFPE tissue samples) in a background of wild-type DNA. Mutant DNA was 2-fold diluted, using 10 ng as initial amount. Total amount of DNA (mutant plus wild-type) was maintained in 25 ng per well.

The limit of detection was considered as the dilution that shows a statistically significant difference from the negative controls or the lowest mutant concentration detected where the lower error bar of the measured mutant concentration does not overlap with the upper error bar of the measured mutant concentration in the wild-type-only (mutation-negative) control. Taking this into consideration, the mutation G719S could be detected by ddPCR even 0.058% mutant fraction (Figure 1B).

### Threshold Setting for Detection of True Positives Results

**Figure 2:**
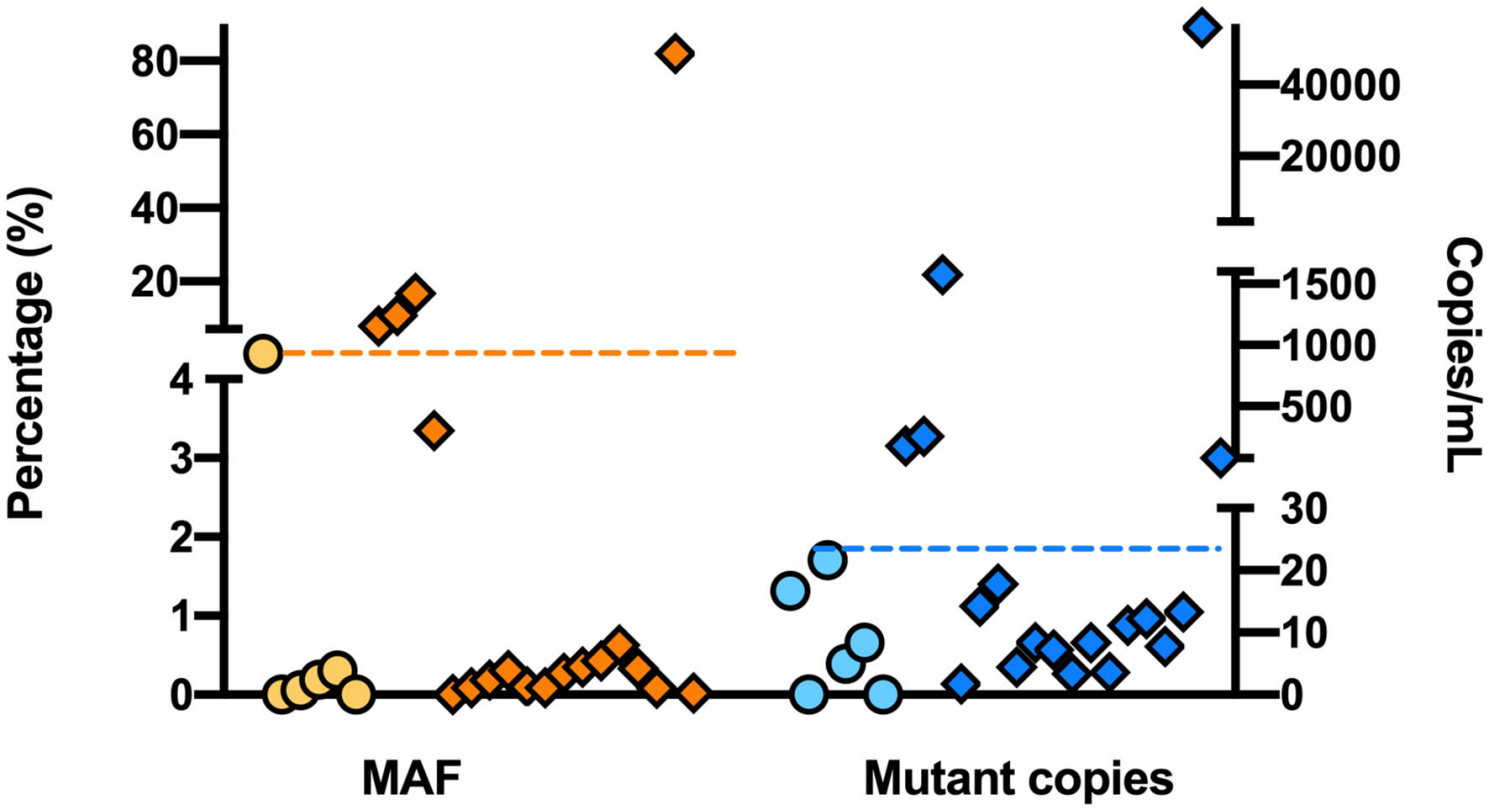
Detection of G719S in a healthy population using ddPCR. Concentration is represented in copies per milliliter of plasma in both healthy (∙) and patient (♦) groups; where dashed line represents a candidate threshold for positive results with high sensitivity.

To optimize the specificity of the *EGFR* genotyping assay, we tested the incidence of false-positive reads in a healthy population of six volunteers. At least, we performed six independent reactions for each individual. Low levels of *EGFR* G719S were detected in healthy volunteers with a peak level of 21.65 copies/mL (Figure 2) and 6.35 MAF. Using 22 mutant copies/mL as threshold for a positive result and MAF of 6.5%, five of 22 patients included in the study were real G719S plasma-positive patients.

### Quantifying Mutant Load

**Figure 3:**
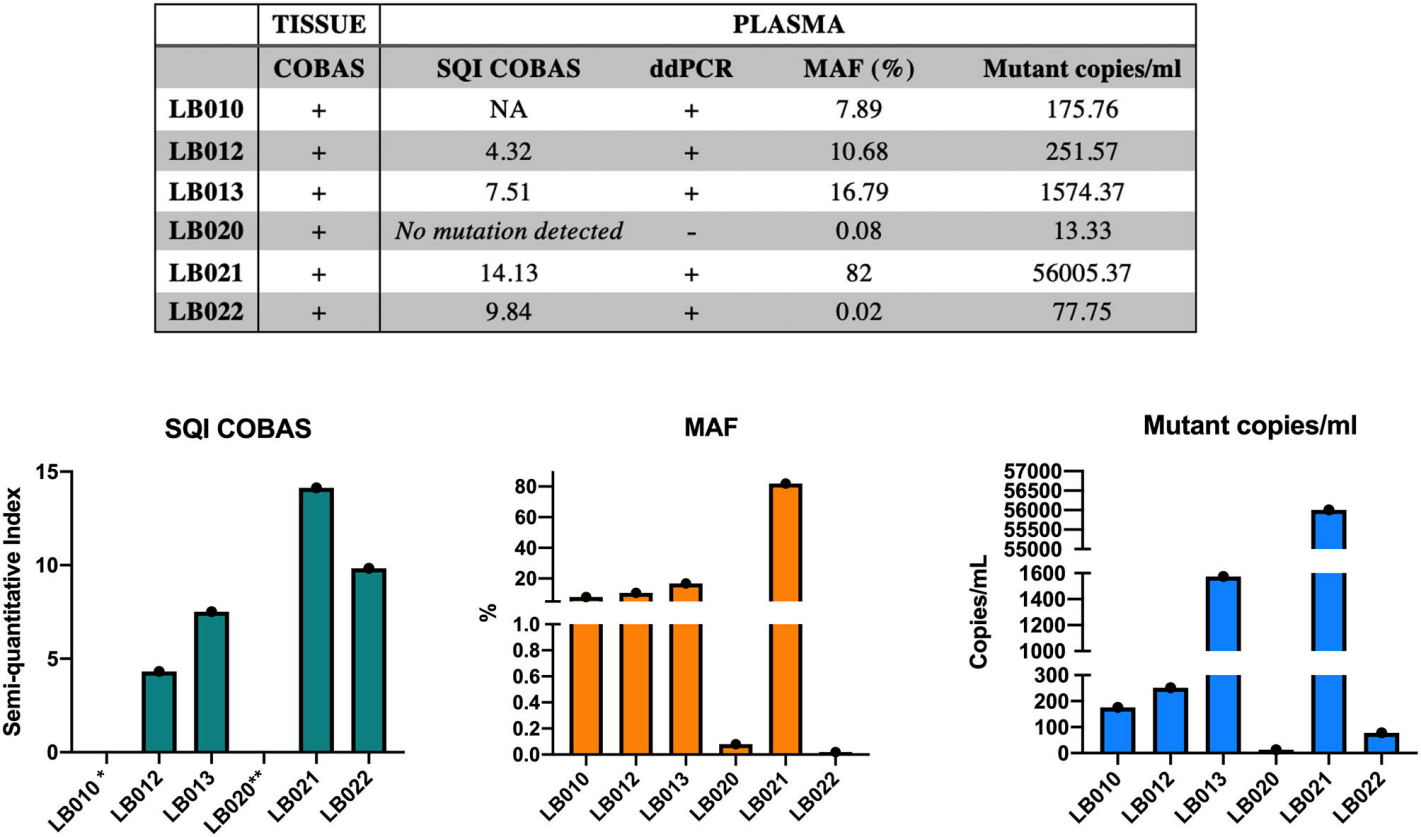
Summary of results obtained by COBAS and ddPCR and comparative evaluation for G719S detection in plasma. SQI, Semiquantitative Index obtained by COBAS® 4800 System. *For sample LB010 SQI was not available. **For sample LB020 no mutation was detected in plasma using COBAS (SQI).

Once a threshold and the sensitivity ddPCR for G719S mutation detection was stablished, MAF and mutant copies of G719S in plasma samples were calculated (Figure 3). It was observed that the patients whose tissue biopsy was G719S positive as assessed by COBAS system, mutation was also detected in plasma with COBAS and ddPCR. However, in the case of the three patients recruited from HGUVA in which the tissue specimen was tested with a different technology (Therascreen EGFR kit, Qiagen) there was one of the plasma samples (LB020) for which ddPCR and COBAS tested negative with some level of detection below the threshold.

Overall, higher values of Semiquantitative Index (SQI) obtained by COBAS System correlated with higher MAF and mutant copies/ml obtained by ddPCR. As agreement measure of both techniques used in the present work, it was calculated the Kappa coefficient (K = 0.88), which indicated a highly positive correlation between COBAS and ddPCR results.

## Discussion

Targeted analysis for pathogenic variants in driver genes is the most promising approach for choosing personalized and more effective treatments to NSCLC patients. The number of FDA approved drugs targeting NSCLC driver genes has increased during the last decade. But routinely, only the most common mutations are studied. However, there are rarer mutations which also contribute to tumor heterogeneity that can also be attacked, as G719S and L851Q mutations in *EGFR*. Moreover, in recent years, liquid biopsy has been introduced as a tool of high potential for obtaining samples noninvasively of cancer patients in order to carry out this genetic diagnosis. Several technologies have been developed for the study of circulating tumor DNA, among which the ddPCR provides greater accuracy, sensitivity and absolute quantification in comparison to other conventional techniques used to date.

We herein describe the development of ddPCR technique for G719S rare mutation detection in advanced NSCLC patients using plasma samples. From a technical point of view, the ability to discriminate mutant sequences from wild-type is one of the critical steps of the study. The separation of the signal can be affected by both concentration DNA input and cross-reaction of probes to detect mutation and native sequence. For this reason, we performed temperature and concentration gradients using G719S positive FFPE samples to determine the conditions under which probes and primers work more efficiently in order to minimize false positive results. Because circulating tumor DNA represents 0.1% or even less of total circulating DNA (18), the sensitivity was evaluated by concentration gradients and using DNA from tissue samples. In our population, we had been able to detect G719S mutation even a dilution of 0.058%. This result is in agreement with those presented by Oxnard et al. (19) and Zhu et al. (20) who also determined ddPCR as a highly sensitive technique showing >80% sensitivity when evaluating L858R and exon 19 deletion mutations.

The strategy of using healthy controls to test false positive results and to stablish a threshold to consider a result as true positive result has previously used by other groups (21–23). In the present study we used blood samples from six healthy people and the threshold was set in 22 mutant copies/mL as and MAF of 6.5%. At this point, it is important to note that LB020 ddPCR results showed positive signal for G719S, but in this context cannot be considered as positive in plasma samples because mutation has not been detected by SQI (COBAS) and does not exceed the applicable threshold that was determined previously. One possible explanation is that LB020 tumor subpopulation carrier of G719S mutation was too small to exceed the threshold stablished and to be detectable through ddPCR and COBAS in plasma samples, respectively. This is a real liquid biopsy situation in which subclones harboring a specific mutation could be represented in a low concentration in cell-free DNA only providing limited signals when assessed by digital PCR.

Despite sample size limitations, the present work shows a robust way to detect G719S mutation in NSCLC patients by ddPCR. However, it should be taken into account that if larger population could be tested, thresholds and correlations calculated may undergo slight variations. Thus, as more NSCLC patients with G719S mutation are detected in the Hospital, it would be advisable to include them into this study to validate the results.

One of the advantages of digital approaches is the quantification without the need for a standard curve. Taking advantage of this capacity, mutant allele load was calculated for three patients whose genetic diagnosis was positive for G719S in tissue biopsy. Also, the obtained values in plasma samples by ddPCR were compared with an approved genotyping methodology in clinical routine, COBAS 4800 System. We could observe that mutation studied values obtained by ddPCR corresponded with positive values in tissue biopsy using COBAS System. These results are comparable to those obtained by Zhu and Weber et al. which show a 90% of concordance between plasma and tissue determination in other *EGFR* mutations with K values of 0.75 and 0.62, respectively (20, 24). Taking in consideration that in the current study sample size is limited, our results are in the same line as those published previously. In terms of correlation between COBAS and ddPCR in plasma samples, it has been shown higher rates obtained by COBAS correlates with greater mutant load in ddPCR that is statistically significant.

Until today, several studies have addressed a comparative analysis between digital and non-digital platforms. In general, digital techniques show greater sensitivity than non-digital techniques. This may be because, as detailed in the COBAS *EGFR* mutation test guide, the system is only capable of detecting mutations with a sensitivity of 5% (25). More specifically, as shown by the results of Thress et al. and Watanabe et al. ddPCR is one of the most sensitive techniques for genotyping ctDNA (26, 27). In the present work, digital and non-digital platforms have been compared to evaluate G719S mutation detection when using tissue and liquid biopsy. Similar results were published by Kim et al., when comparing ddPCR and COBAS platforms for EGFR mutation detection using formalin-fixed paraffin embedded (FFPE) biospecimens to address clinical and quality control issues (28). In the current scenario and due to the wide range of analytical techniques, laboratories will be able to select the optimal platform for their needs.

This research focuses on the development of G719S mutation detection using ddPCR in patients with advanced NSCLC without using commercial primers. Results obtained in the current study suggest ddPCR as a sensitive, specific and low cost genotyping tool for lung cancer patients and could also be applied to other cancers. That is why, if results are validated, the analysis of the mutational status of *EGFR*, specifically G719S mutation, could result in a new biomarker in NSCLC and could join gradually in clinical practice.

## Data Availability Statement

The raw data supporting the conclusions of this article will be made available by the authors, without undue reservation.

## Ethics Statement

The studies involving human participants were reviewed and approved by Clinical Research Ethics Committee of the Balearic Islands (CEIC-IB). The patients/participants provided their written informed consent to participate in this study.

## Author Contributions

AO-H, CG-B, MG, AA, RM, VA, and JT contributed conception and study design. ÚS, AB, and SC-F contributed to sample collection. ME-S (1st author) and ME-S (2nd author) performed the experiments and organized the database. ME-S (1st author) analyzed data, plotted the results, and performed the statistical analysis. ME-S (1st author) and AO-H wrote the first draft of the manuscript. All authors contributed to manuscript revision and read and approved the submitted version.

## Conflict of Interest

The authors declare that the research was conducted in the absence of any commercial or financial relationships that could be construed as a potential conflict of interest.
