# Supplementary material for: Detection of the EGFR G719S Mutation in Non-small Cell Lung Cancer Using Droplet Digital PCR
Source: Front Med (Lausanne). 2020 Nov 13;7:594900. doi: 10.3389/fmed.2020.594900 (PMC7691481; doi:10.3389/fmed.2020.594900)
Supplement: Supplementary file 1 [file Table_1.DOCX]

# Suplementary material

Table S1. Primers and probe sequences for ddPCR assays

| ID | Sequence |
| --- | --- |
| *EGFR* G719S-Fw | TGGAGAAGCTCCCAACCAA |
| *EGFR* G719S-Rev | CTTATACACCGTGCCGAAC |
| *EGFR* G719S-FAM | TGCTG**A**GCTCCGGTGC |
| *EGFR* G719S-HEX | TGCTG**G**GCTCCGGTGC |
